# Hypoxia-Inducible Factor 1A Upregulates HMGN5 by Increasing the Expression of GATA1 and Plays a Role in Osteosarcoma Metastasis

**DOI:** 10.1155/2019/5630124

**Published:** 2019-12-16

**Authors:** Enjie Xu, Zhe Ji, Heng Jiang, Tao Lin, Jun Ma, Xuhui Zhou

**Affiliations:** ^1^Department of Orthopedics, Changzheng Hospital, Second Military Medical University, Shanghai, China; ^2^Department of Orthopedics, People's Hospital of Xinjiang Uygur Autonomous Region, Xinjiang, China

## Abstract

Osteosarcoma is one of the most common malignant tumors in children and adolescents and is characterized by early metastasis. High-mobility group N (HMGN) domains are involved in the development of several tumors. Our previous study found that HMGN5 is highly expressed in osteosarcoma tissues and knockdown of HMGN5 inhibits migration and invasion of U-2 OS and Saos-2 cells. A hypoxic environment is commonly found in solid tumors such as osteosarcoma and is likely to be associated with tumor metastasis, so we further explored the relationship between HMGN5 and the hypoxic environment. Hypoxia-inducible factor 1A (HIF1A) is an adaptive factor in the hypoxic environment. We found that HIF1A and HMGN5 were upregulated in osteosarcoma (OS) cells cultured in the hypoxic environment, and the results of overexpression and knockdown experiments showed that HIF1A upregulated the transcription factor GATA1 and further promoted the expression of HMGN5. In addition, MMP2 and MMP9 were subsequently upregulated through the c-jun pathway, and finally, this promoted the migration and invasion of OS cells. It is suggested that HMGN5 may be an important downstream factor for HIF1A to promote osteosarcoma metastasis. It has an important clinical significance for the selection of therapeutic targets for osteosarcoma.

## 1. Introduction

Osteosarcoma is a highly malignant bone tumor disease derived from mesenchymal cells and is one of the most common malignant tumors in children and adolescents [1, 2]. It tends to metastasize at an early stage. When osteosarcoma is diagnosed, about 15–20% of patients already have macroscopic evidence of metastases, most commonly in the lungs (85–90%), sometimes in the bone (8–10%), and occasionally in the lymph nodes [3–5]. The main treatments are still tumor resection and nonspecific combination chemotherapy [6–9]. With the development of treatment of primary tumor, the long-term survival rate of osteosarcoma patients has increased [10], but the five-year survival rate of patients with distant metastases is still low (0–29%) [11, 12]. In addition, fast-growing solid tumors such as osteosarcoma often have a hypoxic microenvironment inside, which further promotes the metastasis of osteosarcoma [10].

HIF1A is a key factor in the hypoxic microenvironment and is closely related to the expression of various tumor-associated factors. Meanwhile, HMGN5 has been shown to be associated with metastasis of tumors [12]. Our previous study found that HMGN5 is highly expressed in osteosarcoma tissues and knockdown of HMGN5 inhibits migration and invasion of U-2 OS and Saos-2 cells [13].

We speculated that HMGN5 may be a downstream factor of HIF1A which plays an important role in osteosarcoma metastasis under hypoxic conditions. Therefore, we plan to explore the relationship between HIF1A and HMGN5 in a hypoxic environment and its effect on osteosarcoma metastasis.

## 2. Materials and Methods

Our research team has established standardized experimental procedures for cell culture, viral transfection, RT-qPCR, western blot, etc. The process of these assays in this article is similar to that of our previous paper [14].

### 2.1. Cell Culture

DMEM (Hyclone, Utah, USA) medium contains 10% FBS (FBS, Gibco, NY, USA), penicillin (100 U/L, Invitrogen, NY, USA), and streptomycin (100 mg/L, Invitrogen, NY, USA). McCoy's 5a (Invitrogen, NY, USA) medium contains 15% FBS (FBS, Gibco, NY, USA), penicillin (100 U/L, Invitrogen, NY, USA), and streptomycin (100 mg/L, Invitrogen, NY, USA). U-2 OS osteosarcoma cells were cultured in DMEM and placed in a humidified atmosphere of 5% CO_2_ at 37°C. Saos-2 osteosarcoma cells were cultured in McCoy's 5a medium and placed in a humidified atmosphere of 5% CO_2_ at 37°C. We divided the cells into two groups: a normoxic group and a hypoxic group. The normoxic group was cultured in 20% O_2_, and the hypoxic group was cultured in 1% O_2_. All experimental results from U-2 OS cells were repeated in Saos-2 cells.

### 2.2. Overexpression of HIF1A and Knockdown of HMGN5 and GATA1

Recombinant lentiviruses overexpressing HIF1A and blank lentivirus were purchased from Shanghai GeneChem Co., Ltd. (Shanghai, China), and OS cells were transfected with overexpression lentiviruses (HIF1A^OE^) and blank lentivirus (Cherry).

Recombinant lentiviruses knocking down HMGN5 and GATA1 were also purchased from Shanghai GeneChem Co., Ltd. (Shanghai, China), and HIF1A-overexpressed OS cells were transfected with knockdown lentiviruses (HMGN5^sh^ or GATA1^sh^ and blank lentivirus (GFP, Table 1)).

We determined that the optimal multiplicity of infection (MOI) for U-2 OS virus infection was 10 by preexperiment and MOI for Saos-2 was 15. We conducted the formal experiments based on these MOI values. In order to increase the proportion of infected cells, we performed flow sorting after 3 days of cell infection and continued to culture the cells with cherry or green fluorescence. We extracted RNA and proteins when the cells reached 70–80% confluence. Overexpression and knockdown efficiency were confirmed by RT-qPCR and western blot.

### 2.3. RT-qPCR

RT-qPCR was applied to quantitatively determine the expression level of HIF1A, HMGN5, and GATA1 using the SYBR-Premix Ex Taq (Takara, Japan) and ABI Prism 7900HT sequence detection system (Applied Biosystems, Carlsbad, CA). Total RNA of each group was extracted with TRIzol according to the manufacturer's instructions. The mRNA was transcribed into cDNA using a Reverse Transcription kit (Applied Biosystems, Carlsbad, CA). The genes were amplified using specific primers, and human *β*-actin gene was used as an endogenous control. The PCR primer sequences used were as follows: HIF1A: forward: 5′-GAACGTCGAAAAGAAAAGTCTCG-3′, reverse: 5′-CCTTATCAAGATGCGAACTCACA-3′; HMGN5: forward: 5′-CAGGTCAAGGTGATATGAGGCA-3′, reverse: 5′-GCTTGGGCACTTGTATCTATGT-3′; GATA1: forward: 5′-CTGTCCCCAATAGTGCTTATG-3′, reverse: 5′-GAATAGGCTGCTGAATTGAGGG-3′; *β*-actin: forward: 5′-ACCGAGCGCGGCTACAG-3′, reverse: 5′-CTTAATGTCACGCACGATTTCC-3′. Data were analyzed using the comparative Ct method (2^−ΔΔCt^). We repeated the RT-qPCR experiment three times with the same samples and the same primers.

### 2.4. Western Blot Assay

HMGN5 and GATA1 are mainly expressed in the nucleus, and HIF1A is mainly expressed in the nucleus and cytosol (https://compartments.jensenlab.org). Nuclear proteins were extracted using a NE-PER Nuclear Extraction Reagents (Thermo Fisher Scientific, MA, USA) and whole proteins were extracted using a protein extract kit (Beyotime, Shanghai, China), and the concentration was determined by using a BCA Protein Assay Kit (Pierce). Proteins were run on SDS-PAGE gels and transferred onto polyvinylidene fluoride (PVDF, Millipore, MA, USA) membranes and blocked in 5% bovine serum albumin (BSA, Sigma-Aldrich, St. Louis, MO, USA). Membranes were probed overnight at 4°C with the appropriate primary antibody. Antibody were used as follows: HIF1A (1 : 1000 dilution, ab51608, Abcam, Cambridge, UK), HMGN5 (1 : 2000 dilution, ab186001, Abcam), GATA1 (1 : 500 dilution, ab28839, Abcam), c-jun (1 : 1000 dilution, ab40766, Abcam), MMP2 (1 : 500 dilution, ab97779, Abcam), MMP9 (1 : 1000 dilution, ab76003, Abcam), MMP16 (1 : 500 dilution, ab73877, Abcam), LMNB1 (1 : 10000 dilution, ab16048, Abcam), and *β*-actin (1 : 10000 dilution, ab8226, Abcam). Membranes were then probed with a horseradishperoxidase-conjugated secondary antibody for 1 hour at room temperature. The immunoreactive bands were visualized using an enhanced chemiluminescence (ECL) detection system (No. 32106, Pierce). We chose loading control according to Abcam recommendation (http://www.abcam.com/primary-antibodies/loading-control-guide). The relative protein level in different cell lines was normalized to LMNB1 (Lamin B1) or *β*-actin concentration. Three separate experiments were performed for each group.

### 2.5. Wound-Healing Assay

OS cells were plated in 6-well culture plates and grown to 90% confluence. Wounds were created using a 200 *μ*l micropipette tip. The migration of cells towards the wound was monitored and photographed. Calculated cell migration rate = (0 h width − 24 h or 48 h width)/0 h width × 100%.

### 2.6. Transwell Invasion Assay

The invasive ability of cells was determined in a 6.5 mm transwell (Coring, NY, USA). The filter of the top chamber was matrigel-coated with 50 *μ*l of diluted matrigel, and the lower chamber was filled with 500 *μ*l of DMEM containing 10% FBS. Cells were resuspended in serum-free medium and added to each top chamber. The cells were cultured for 24 h, and the noninvading cells were removed. Invading cells were fixed with 4% paraformaldehyde for 30 minutes and then stained with crystal violet staining solution (Sigma-Aldrich). The number of invading cells were counted and analyzed.

### 2.7. Statistical Analysis

**Figure 1:**
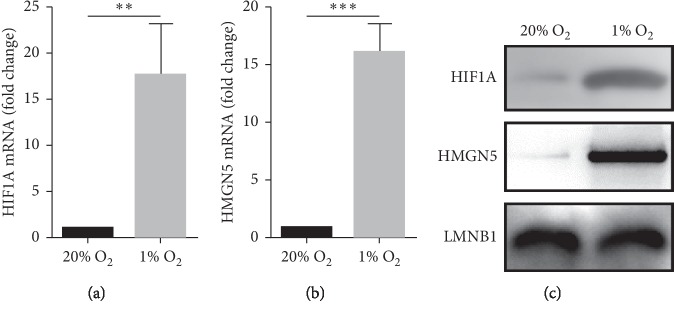
Expression levels of HIF1A and HMGN5 in hypoxic cells were higher than in normoxic cells. (a, b) RT-qPCR showed that the expression levels of HIF1A mRNA and HMGN5 mRNA were higher in hypoxic OS cells. (c) Western blot showed that the expression levels of HIF1A protein and HMGN5 protein were higher in hypoxic OS cells. Data were presented as the mean ± SD. ^*∗∗*^*P* < 0.01; ^*∗∗∗*^*P* < 0.001.

**Figure 2:**
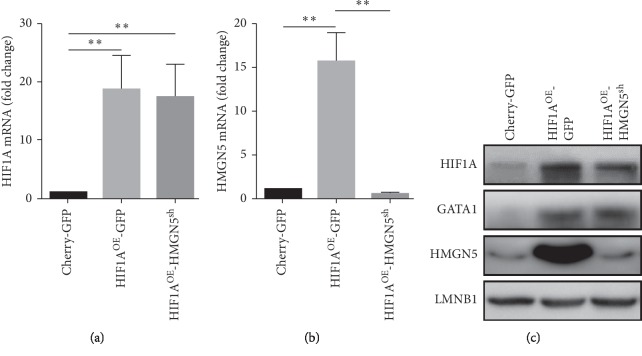
HIF1A upregualtes HMGN5. (a) The expression levels of HIF1A mRNA were higher in the HIF1A^OE^-GFP group and HIF1A^OE^-HMGN5^sh^ group than in the Cherry-GFP group. (b) The expression level of HMGN5 mRNA in the HIF1A^OE^-GFP group was significantly higher than in the Cherry-GFP group and was knocked down in the HIF1A^OE^-HMGN5^sh^ group. (c) The expression levels of HIF1A and GATA1 were higher in HIF1A^OE^-GFP and HIF1A^OE^-HMGN5^sh^ groups than in the Cherry-GFP group. The expression level of HMGN5 in the HIF1A^OE^-GFP group was higher than in the Cherry-GFP group and was knocked down in the HIF1A^OE^-HMGN5^sh^ group. Data were presented as the mean ± SD. ^*∗∗*^*P* < 0.01.

**Figure 3:**
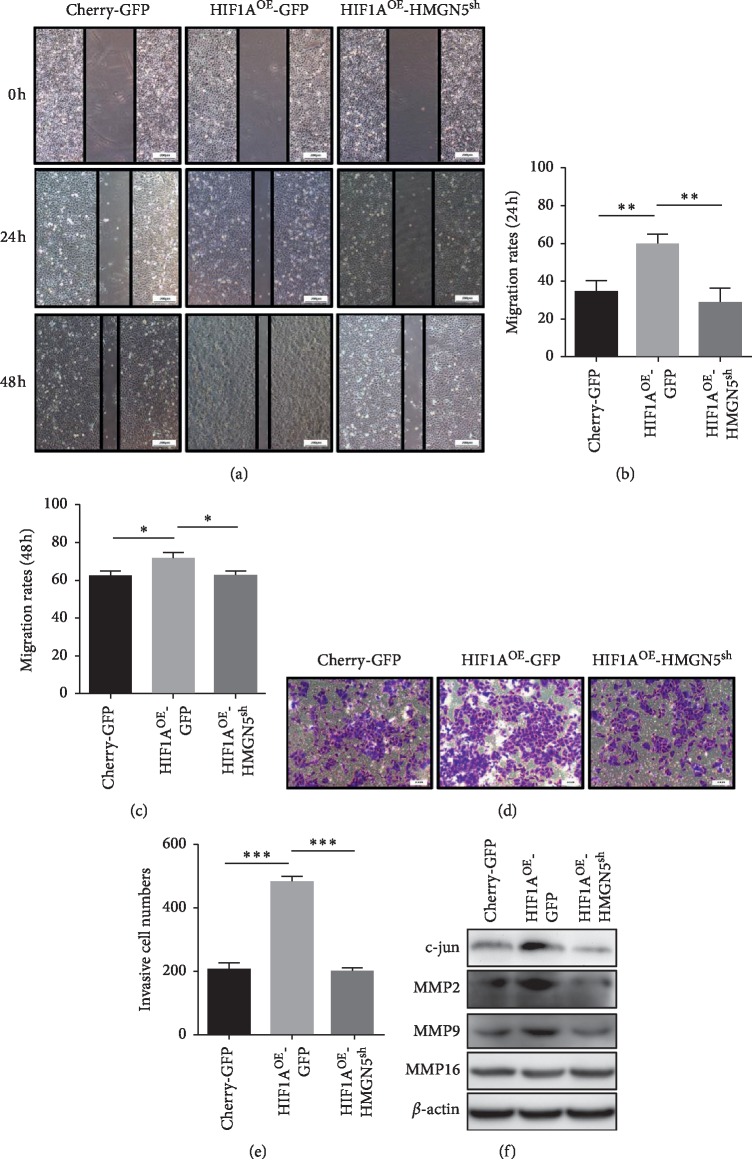
Reversal of enhanced migration and invasion of osteosarcoma cells under hypoxic conditions by knocking down HMGN5. (a) Wound-healing assay in 24 hours and 48 hours for each group. (b, c) Statistical analysis of cell migration rate of each group in 24 hours and 48 hours; the results showed that the migration ability of the HIF1A^OE^-GFP group was significantly enhanced compared with the Cherry-GFP group, while the migration ability of the HIF1A^OE^-HMGN5^sh^ group was significantly lower than that of HIF1A^OE^-GFP group. (d) Transwell invasion assay for each group. (e) Statistical analysis of invading cells of each group; the results showed that the invasion ability of the HIF1A^OE^-GFP group was significantly enhanced compared with the Cherry-GFP group, while the invasion ability of the HIF1A^OE^-HMGN5^sh^ group was significantly lower than that of the HIF1A^OE^-GFP group. (f) The expression levels of c-jun, MMP2, and MMP9 in the HIF1A^OE^-GFP group were higher than in Cherry-GFP group. The expression levels of c-jun, MMP2, and MMP9 in the HIF1A^OE^-HMGN5^sh^ group were lower than in the HIF1A^OE^-GFP group. Data were presented as the mean ± SD. ^*∗*^*P* < 0.05; ^*∗∗*^*P* < 0.01; ^*∗∗∗*^*P* < 0.001.

**Figure 4:**
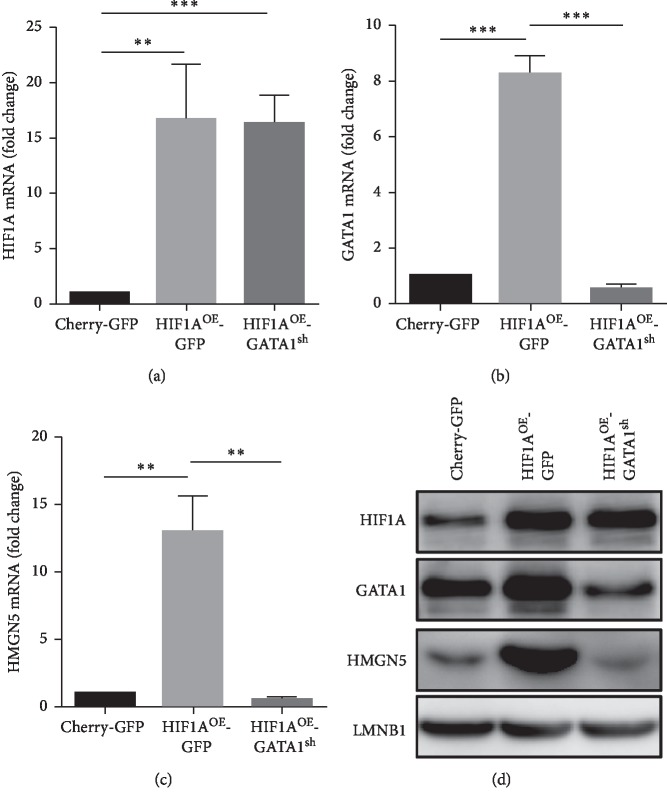
HIF1A upregulates GATA1 to promote the expression of HMGN5. (a) The expression levels of HIF1A mRNA were higher in HIF1A^OE^-GFP and HIF1A^OE^-GATA1^sh^ groups than in the Cherry-GFP group. (b) The expression level of GATA1 mRNA in the HIF1A^OE^-GFP group was significantly higher than in the Cherry-GFP group and was knocked down in the HIF1A^OE^-GATA1^sh^ group. (c) The expression level of HMGN5 mRNA in the HIF1A^OE^-GFP group was significantly higher than in the Cherry-GFP group, and the expression level of HMGN5 mRNA in HIF1A^OE^-GATA1^sh^ group was significantly lower than in the HIF1A^OE^-GFP group. (d) GATA1 and HMGN5 had higher expression levels in the HIF1A^OE^-GFP group (compared with Cherry-GFP group) but lower expression in the HIF1A^OE^-GATA1^sh^ group (compared with HIF1A^OE^-GFP group). Data were presented as the mean ± SD. ^*∗∗*^*P* < 0.01; ^*∗∗∗*^*P* < 0.001.

Three separate experiments were performed for each experiment. Analysis of the data was performed using SPSS version 23.0, with *P* value <0.05 considered statistically significant. All data were presented as the mean ± SD. The Mann–Whitney *U* test was used to compare the difference between two groups (Figures 1(a) and 1(b)). The Kruskal–Wallis *H* test was used to analyze the differences between three groups (Figures 2(a), 2(b), 3(b), 3(c), 3(e), and 4(a)∼4(c)).

## 3. Results

### 3.1. High Expression of HIF1A and HMGN5 in Hypoxic Environment-Cultured OS Cells

We cultured OS cells in 20% O_2_ (normoxic group) and 1% O_2_ (hypoxic group). The results of RT-qPCR and western blot showed that the expression level of HMGN5 was higher in hypoxic OS cells (Figures 1(b) and 1(c)). It suggests that some hypoxia-related factors may upregulate the expression of HMGN5. HIF1A is an adaptive factor that is highly expressed in the hypoxic environment (Figures 1(a) and 1(c)), which is closely related to the expression of various tumor-associated factors. We subsequently did some work to clarify the relationship between HIF1A and HMGN5.

### 3.2. HIF1A Was Overexpressed and HMGN5 Was Knocked Down Successfully in OS Cells

We overexpressed HIF1A and knocked down HMGN5 in OS cells, and finally, we have 3 groups of cells to explore the relationship between HMGN5 and HIF1A: Cherry-GFP group (control), HIF1A^OE^-GFP group (HIF1A overexpression), and HIF1A^OE^-HMGN5^sh^ group (HIF1A overexpression and HMGN5 knockdown) (Table 1). RT-qPCR results showed that HIF1A was overexpressed in the HIF1A^OE^-GFP group and HIF1A^OE^-HMGN5^sh^ group, and HMGN5 was knocked down in the HIF1A-HMGN5^sh^ group (Figures 2(a) and 2(b)). The results of western blot further confirmed the overexpression and knockdown (Figure 2(c)).

### 3.3. Knockdown of HMGN5 Inhibits Migration and Invasion of OS Cells under Hypoxic Conditions

The role of HIF1A and HMGN5 in the migration and invasion of OS cells was determined using wound-healing and transwell invasion assay. The results showed that the migration and invasion of the HIF1A^OE^-GFP group were significantly enhanced compared with the Cherry-GFP group, while the migration and invasion of the HIF1A^OE^-HMGN5^sh^ group were significantly lower than those of the HIF1A-GFP group. The results showed that overexpression of HIF1A in OS cells significantly increased cell migration and invasion, while knockdown of HMGN5 reversed these effects (Figures 3(a)–3(e)), suggesting that HIF1A enhances cell migration and invasion partially through HMGN5 in OS cells.

Previous studies have confirmed that HMGN5 regulates the expression of MMP2 and MMP9 via c-jun and then contributed to the migration and invasion of clear cell renal cell carcinoma (ccRCC) cells [15]. Our results showed that upregulated HMGN5 in a hypoxic environment increased the expression of c-jun and finally increased the expression of MMP2 and MMP9, which was associated with migration and invasion of osteosarcoma (Figure 3(f)).

### 3.4. HIF1A Regulates the Expression of HMGN5 through the Transcription Factor GATA1

HIF1A upregulated the expression of HMGN5 mRNA (Figure 2(b)). We predicted transcription factors binding to the HMGN5 promoter by PROMO transcription factor prediction software and found that GATA1 is probably an important transcription factor of HMGN5. Western blot confirmed that HIF1A upregulates GATA1 expression (Figure 2(c)). We obtained a new group through lentiviral infection named “HIF1A^OE^-GATA1^sh^ group” (HIF1A overexpression and GATA1 knockdown, Figures 4(a) and 4(b)) to confirm that HIF1A regulates HMGN5 through GATA1. The results of RT-qPCR and western blot showed that overexpression of HIF1A upregulated GATA1 and HMGN5, while knockdown of GATA1 reduced the expression of HMGN5 (Figures 4(a)–4(d)).

## 4. Discussion

It is estimated that 20% of patients with osteosarcoma have metastases at the time of initial diagnosis [16], and more than 80% of patients with osteosarcoma develop pulmonary metastases within two years and, if left untreated, die of disease within a few months [17]. The main treatments for osteosarcoma are chemotherapy and surgical intervention. However, a significant proportion of these patients eventually develop pulmonary metastases and succumb to their disease even after chemotherapy and surgical excision [18–20]. Despite complete tumor resection and intensive chemotherapy, approximately 30% to 40% of patients with osteosarcoma relapse [21–25], and recurrence of osteosarcoma is the most common in the lungs [26]. Therefore, there is a need to develop new and safe approaches to the treatment of osteosarcoma metastasis.

Osteosarcoma is a solid malignant tumor characterized by rapid growth and a high rate of metastasis [27]. It increases in a short period of time and leads to an oxygen-deficient environment inside the tumor [10]. Hypoxia has been shown to play important roles in the progression and metastasis of many cancers such as breast cancer [28], colon cancer [29], and melanoma [30]. HIF is a key regulator of local and systemic responses to hypoxia that occur during normal development and pathological/physiological processes [31]. HIF1A stability is regulated at the posttranslational level by oxygen concentration, which is continuously degraded under normoxia conditions. The activity of prolyl hydroxylases is diminished under hypoxic conditions since they require ferrous ions and O_2_ for their function, which leads to HIF1A stabilization [32]. Overexpression of HIF1A has been reported to indicate poor prognosis in osteosarcoma patients [33]. In our study, we found that HIF1A upregulated HMGN5 and further promoted migration and invasion of OS cells.

PROMO transcription factor prediction software was used to find that GATA1 is probably a transcription factor of HMGN5. Our results showed that overexpression of HIF1A promoted the expression of HMGN5 by upregulating GATA1. GATA1 was originally identified in 1989 as a transcription factor that binds to the *β*-globin regulatory region, and it was later revealed to be a member of the GATA transcription factor family [34–36]. GATA1 is widely expressed in human and mammalian cells [37, 38]. Researchers found that GATA1 plays an important role in blood cell differentiation [39–41]. Zhang et al. found that the upregulation of GATA1 during hypoxia is directly mediated by HIF1. The mRNA expression of some erythroid differentiation markers were increased under hypoxic conditions but decreased with RNA interference of HIF1A or GATA1 [42].

The high-mobility group (HMG) includes HMGA, HMGB, and HMGN. Previous studies have confirmed that HMGA and HMGB are related to tumor metastasis. In recent years, the function of HMGN in tumor invasion and metastasis has been extensively studied [12]. Studies have shown that HMGN5 plays an important role in the metastasis of prostate cancer [43], bladder cancer [44, 45], kidney cancer [46], and breast cancer [47]. Our previous study found that HMGN5 is highly expressed in osteosarcoma tissues, and knockdown of HMGN5 inhibits migration and invasion of U-2 OS and Saos-2 cells [13]. HMGN5 plays a role in PI3K/AKT signaling pathway or MAPK/ERK signaling pathway [12]. In addition, Ji et al. found that the expression level of HMGA1 mRNA and protein were significantly higher in the hypoxic environment [48]. In our study, we found that overexpression of HIF1A upregulated the expression of transcription factor GATA1 and further promoted the expression of HMGN5. The higher expression of HMGN5 upregulated MMP2 and MMP9 through the c-jun pathway and finally promoted the migration and invasion of OS cells. It is suggested that HMGN5 is an important downstream factor for HIF1A to promote osteosarcoma metastasis. It has an important clinical significance for the selection of therapeutic targets for osteosarcoma.

This is a limited research because we did not repeat the experiment in animal models. In vivo assays are necessary to confirm our in vitro results, but it is very difficult to raise animals for a long time under hypoxic conditions due to the limitations of research facilities. In the next step, we plan to explore the effects of HIF1A and HMGN5 on osteosarcoma cell metastasis by injecting three groups of cells (Cherry-GFP, HIF1A^OE^-GFP, and HIF1A^OE^-HMGN5^sh^) into the tail vein of nude mice.

## 5. Conclusion

We found that HIF1A upregulated the transcription factor GATA1 and further promoted the expression of HMGN5. In addition, MMP2 and MMP9 were subsequently upregulated through the c-jun pathway, and this finally promoted the migration and invasion ability of osteosarcoma cells. It is suggested that HMGN5 may be an important downstream factor for HIF1A to promote osteosarcoma metastasis.

## Figures and Tables

**Table 1:** Cellular virus infection.

Group	Overexpression	Knockdown
Cherry-GFP	—	—
HIF1A^OE^-GFP	HIF1A	—
HIF1A^OE^-HMGN5^sh^	HIF1A	HMGN5
HIF1A^OE^-GATA1^sh^	HIF1A	GATA1

## Data Availability

The data used to support the findings of this study are available from the corresponding author upon request.

## Abbreviations

HIF1A: Hypoxia-inducible factor 1A
HMGN5: High-mobility group nucleosome binding domain 5
GATA1: GATA binding protein 1
OS: Osteosarcoma
MMP2: Matrix metallopeptidase 2
MMP9: Matrix metallopeptidase 9.
